# Medication adherence in epilepsy: the role of adverse drug reactions and patient knowledge-attitude-behavior

**DOI:** 10.1186/s42494-025-00237-z

**Published:** 2026-01-04

**Authors:** Ana Hulliyyatul Jannah, Nadia Devianca, Retnosari Andrajati, Fitri Octaviana, Winnugroho Wiratman, Luh Ari Indrawati, Adrian Ridski Harsono, Manfaluthy Hakim, Ahmad Yanuar Safri, Nurul Fadli, Astri Budikayanti

**Affiliations:** 1 Clinical Pharmacy Department, Faculty of Pharmacy, Universitas Indonesia, Depok, 16424 Indonesia; 2 Neurology Department, Faculty of Medicine, Universitas Indonesia/Dr, Cipto Mangunkusumo National Referral Hospital, Jakarta, 10430 Indonesia

**Keywords:** Epilepsy, Adverse drug reaction, Knowledge, Attitude, Behavior, Medication adherence

## Abstract

**Background:**

Adherence to epilepsy treatment varies greatly and is often compromised by adverse drug reactions. Spontaneous reporting of these reactions has improved pharmacovigilance in many countries. Knowledge, attitudes, and behavior are also key factors influencing treatment adherence. This study aimed to investigate the types of adverse drug reactions in epilepsy, as well as the knowledge, attitudes, and behavior of patients, and how these factors relate to medication adherence.

**Methods:**

This cross-sectional study assessed adverse drug reactions using the Indonesian version of the Liverpool Adverse Event Profile and medication adherence with the Morisky Adherence Questionnaire. Knowledge, attitude and behavior were measured using a self-reported Indonesian knowledge-attitude-behavior questionnaire. Data were collected at two periods of time (2019 and 2022), in which the first period measured adherence, and adverse drug reactions, while the second period measured adherence, and adverse drug reactions, alongside knowledge, attitude, and behavior. Data were gathered from epilepsy patients at the neurology outpatient clinic at Cipto Mangunkusumo National Referral Hospital, and analyzed using Chi-square, likelihood ratio, independent *t*-test, or Mann–Whitney U Tests, followed by multivariate logistic regression.

**Results:**

Adherence rates in both periods exceeded 50% (50.88% vs. 55.70%). Adverse drug reactions were reported by 78.07% of subjects, and were significantly associated with non-adherence (*P* = 0.007). The mean knowledge score was 15.41 ± 3.83, and lower knowledge scores were linked to higher odds of non-adherence. Although not statistically significant (*P* = 0.077), higher knowledge scores showed a trend toward more frequent reporting of adverse drug reactions. Female subjects had higher odds of adherence compared to males (*P* = 0.013).

**Conclusions:**

Our findings suggest that adverse drug reaction reporting, along with patient knowledge, attitudes, and behavior, are important factors associated with medication adherence. Targeted interventions by healthcare providers to support these areas may help improve adherence in people with epilepsy.

## Background

Medication adherence is a major challenge in long-term epilepsy management. Patients with epilepsy (PWE) who have low adherence demonstrated a 21% higher risk of uncontrolled seizures [1, 2]. Low or non-adherence often goes unnoticed and can be mistaken for drug-resistant epilepsy (DRE) [3]. In fact, research on pseudo-intractable epilepsy revealed that 40% of PWE with poor seizure control are non-adherent, and in some cases, this non-adherence may contribute to the development of DRE [4].

Adverse drug reactions (ADRs) are a significant factor affecting medication adherence in epilepsy. Several studies have displayed non-adherence rates of 32.3% and 50% in PWE who experienced ADRs, while other research has shown non-adherence rates ranging from 21.2% to 46.8%, consistently highlighting the strong association between ADRs and non-adherence [5–8].

Another key factor influencing medication adherence in epilepsy is knowledge-attitude-behavior (KAB). Higher knowledge about epilepsy correlates with better adherence, with patients who have higher educational status more likely to follow their treatment plans [7, 9]. In contrast, lower educational status or limited knowledge about epilepsy is often linked to poor adherence. Positive attitudes toward medication foster stronger adherence, while negative beliefs—such as doubts about the necessity of medication and concerns about its harm or adverse reactions—lead to reduced adherence. For instance, an Ethiopian study found that 58.6% of patients with low adherence had significant concerns about adverse drug reactions and the purpose of their medication, while 27.7% believed their medication was unnecessary, contributing to 54.5% displaying negative behaviors toward treatment [10].

Currently, no data exists on medication adherence, ADR or KAB related to anti-seizure medications (ASM) in PWE at Cipto Mangunkusumo National Referral Hospital (CMH) in Indonesia, despite differences in patient characteristics compared to other regions. The risk of therapy failure and ADRs in PWE remains significant, making the detection and evaluation of ADRs, along with ASM adherence, crucial for effective epilepsy management. This study aims to assess ADRs, as well as KAB, in PWE and their relationship to medication adherence levels.

## Methods

This was a cross-sectional study conducted at the Neurology Outpatient Clinic of Cipto Mangunkusumo National Referral Hospital (CMH), Faculty of Medicine, Universitas Indonesia. The study was approved by the Health Research Ethics Committee of the Faculty of Medicine, Universitas Indonesia (No. KET-665/UN2.F1/ETIK/PPM.00.02/2019), and all subjects provided written informed consent before participation. The study adhered to the Declaration of Helsinki.

Data collection occurred in two periods due to the COVID-19 pandemic and healthcare system constraints. Although the same patient cohort was originally intended, certain factors, such as the Indonesian national referral policy for patients with controlled seizures, made it difficult to follow up with the same individuals across both periods. The first period, held from August to October 2019, assessed ADRs and medication adherence. The second period, conducted from August to September 2022, evaluated ADRs, KAB, and medication adherence. During the three-year interval, many previously recruited patients had achieved seizure control and were referred back to their primary healthcare centers, per national policy. Therefore, the participants from the two periods represent different samples but were assessed using consistent inclusion criteria and aligned research instruments. Data were sourced from medical records and the CMH Information System.

The inclusion criteria for the study were: patients aged over 18 years, diagnosed with epilepsy, who had been on the same type of ASM for at least one month, were able to read and respond to questions independently, and who provided informed consent. Exclusion criteria included patients with mental retardation or severe psychiatric disorders, liver disorders, stage V chronic kidney disease, those undergoing hemodialysis, and patients taking hormonal contraceptives or other medications with significant interactions requiring medical intervention to mitigate serious side effects.

The main independent variables were ADRs and KAB, while the dependent variable was medication adherence. Medication adherence was assessed through interviews using the Morisky Adherence Questionnaire (MAQ), which includes four yes/no questions, with scores ranging from 0 to 4 (0 = no, 1 = yes). Patients scoring 0 were classified as adherent, while those scoring 1–4 were considered non-adherent [11, 12]. ADR data were collected using the validated Indonesian version of the Liverpool Adverse Event Profile (LAEP), which contains 19 ADR items rated on a 4-point Likert scale (1 = never, 2 = 3 days in the past month, 3 = 15 days in the past month, 4 = almost every day), yielding a total score range of 19–76, with higher scores indicating more frequent symptom reporting [13, 14]. A score of 2–4 on any of the 19 ADR items indicated the presence of an ADR. Knowledge-attitudes-behavior was evaluated using a self-reported Indonesian version of a KAB questionnaire consisting of 7, 8, and 5 questions, respectively, each rated on a 5-point Likert scale (1 = strongly agree, 5 = strongly disagree). The questionnaire had been validated in a previous study. Total scores were calculated by summing the responses in each category, with lower scores reflecting better KAB [15]. Confounding variables included age, gender, ASM duration, type and number of ASMs, and seizure event. Types of ASMs were classified into the internationally accepted system of traditional or new ASMs, based on the year of market introduction before or after 1990; thus, phenytoin, valproic acid, carbamazepine, and phenobarbital were classified as traditional ASMs [16, 17]. Seizure event was defined as having one or more seizures within the past month.

Data were analyzed using SPSS version 26.0. Demographic and clinical characteristics were reported as percentages. Bivariate analysis was conducted using the Chi-square, likelihood ratio, or Fisher’s exact test for categorical variables, and Mann–Whitney U or independent *t*-test for continuous variables. Multivariate logistic regression was applied to control for ADRs, gender, ASM type, number of ASMs, and seizure event. Variables with a *P*-value < 0.25 in bivariate analysis were included in the logistic regression model, while those with *P*-values > 0.25 were excluded. No artificial intelligence (AI) tools were used throughout all periods of the study.

## Results

We enrolled a total of 175 subjects, with 114 in the first period and 61 different subjects in the second period of the study. The age range of 19–39 years dominated both periods (56.1% vs 62.3%) with a majority being female (59.6% vs 57.4%). Most subjects had been on ASMs for over a year (72.8% vs. 91.8%), used traditional ASMs (39.5% vs. 41.0%), and were on polytherapy regimens (52.6% vs. 52.5%). Additionally, most patients had experienced no seizure events in the past month (57.9% vs. 54.1%) (Table 1).

**Table 1:** Distribution of characteristics of epileptic patients in Dr. Cipto Mangunkusumo Hospital (*n* = 175)

Variables	Categories	First Period Data (*n* = 114)		Second Period Data (*n* = 61)	
		N	Percentage (%)	N	Percentage (%)
Age	19–39 years old	64	56.1	38	62.3
	40–60 years old	40	35.1	18	29.5
	> 60 years old	10	8.8	5	8.2
Gender	Male	46	40.4	26	42.6
	Female	68	59.6	35	57.4
Duration of ASM	< 1 year	31	27.2	5	8.2
	> 1 year	83	72.8	56	91.8
Types of ASM	Traditional	45	39.5	25	41.0
	New	29	25.4	22	36.0
	Combinations	40	35.1	14	23.0
Number of ASM	Monotherapy	54	47.4	29	47.5
	Polytherapy	60	52.6	32	52.5
Seizure Event	No	66	57.9	33	54.1
	Yes	48	42.1	28	45.9
Adherence	No	59	52.6	27	44.3
	Yes	55	47.4	34	55.7
ADRs Incidence	No	25	21.9	11	18.0
	Yes	89	78.1	50	82.0
**Scores**		**Median (min–max)**		**Median (min–max)**	
MAQ		1 (0–4)		0 (0–4)	
LAEP		23 (19–57)		25 (19–61)	

*ASM* Anti-seizure medication, *ADR* Adverse drug reactions, *LAEP* Liverpool Adverse Event Profile, *MAQ* Morisky Adherence Questionnaire, *KAB* Knowledge-Attitudes-Behavior, *SD* Standard deviation

A notable difference in adherence between the two periods was observed. In the first period, 47.4% of patients were adherent, which improved to 55.7% in the second period. The median MAQ score decreased from 1 in period 1 to 0 in period 2, indicating an improvement in adherence (Table 1).

**Fig. 1:**
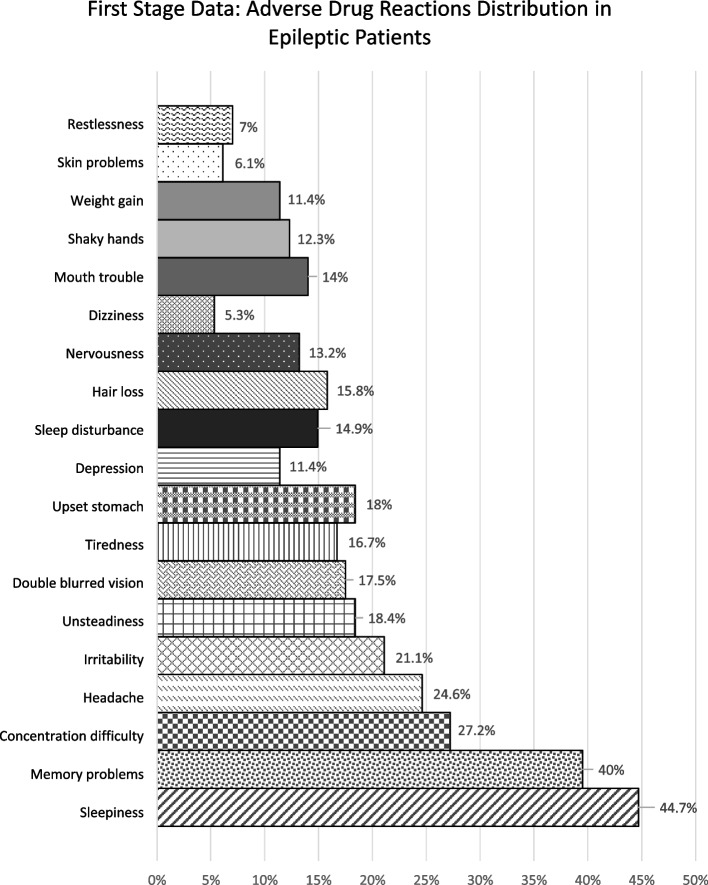
Bar Chart – First Period data: ADR distribution percentage based on LAEP questionnaire of epileptic patients (*n* = 114)

**Fig. 2:**
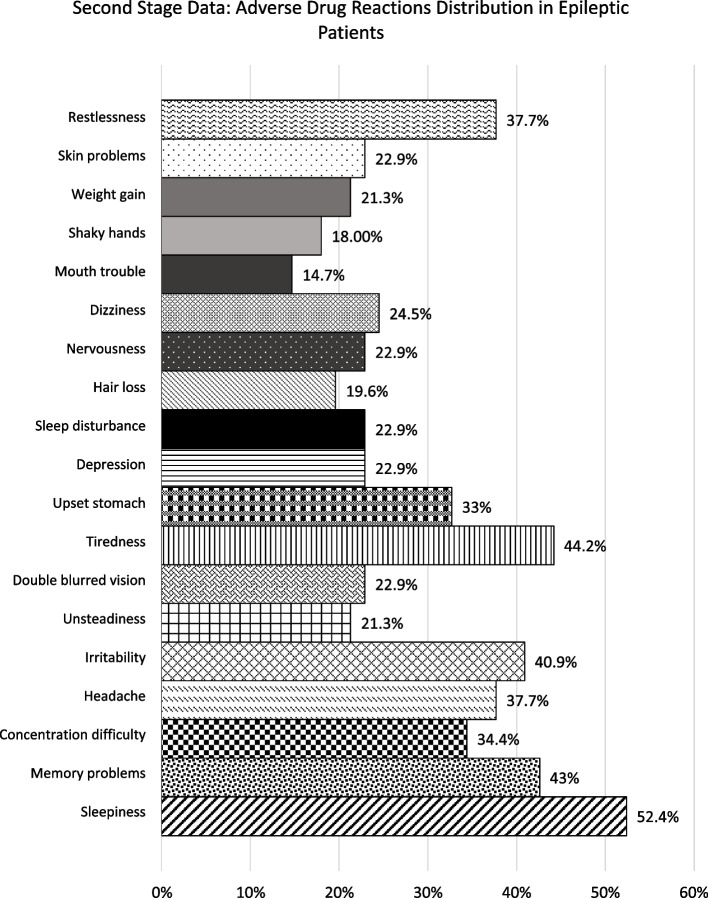
Bar Chart – Second Period data: ADR distribution percentage based on LAEP questionnaire of epileptic patients (*n* = 61)

The incidence of ADRs remained high in both periods (78.1% vs 82.0%). The median LAEP score was 23 (19–57) for the first period and 25 (19–61) for the second (Table 1). The most frequently reported ADRs were sleepiness (44.7% vs. 52.4%), memory problems (40% vs. 42.6%), irritability (21.1% vs. 40.9%), and headaches (24.6% vs. 37.7%). Additional ADRs such as difficulty concentrating were prominent in the first period (27.2%), while tiredness was more frequently reported in the second period (44.2%) (Figs. 1 and 2).

A significant association between ADRs and adherence was observed in the first period (*P* = 0.000), but not during the second period. Other factors, including the type of ASM (*P* = 0.014), number of ASMs (*P* = 0.001), and seizure event (*P* = 0.040), also showed significant associations with adherence in the first period (Table 2).

**Table 2:** Association between ADRs and confounding variables with adherence of epileptic patients

Variables	First Period Data		Second Period Data			
	Adherence Level (*n*%)		*P*-value	Adherence Level (*n*%)		*P*-value
	Non-Adherent	Adherent		Non-Adherent	Adherent	
ADRs			0.000^c^			0.092^c^
Present	52 (92.9)	37 (63.8)		25 (92.6)	25 (73.5)	
Not Present	4 (7.1)	21 (36.2)		2 (7.4)	9 (26.5)	
Age			0.258^b^			0.214^b^
19–39 years old	28 (50)	36 (62.1)		19 (70.4)	19 (55.9)	
40–60 years old	21 (36.5)	19 (32.8)		5 (18.5)	13 (38.2)	
> 60 years old	7 (12.5)	3 (5.1)		3 (11.1)	2 (5.9)	
Gender			0.194^a^			0.037^a^
Male	26 (46.4)	20 (34.5)		16 (59.3)	10 (29.4)	
Female	30 (53.6)	38 (65.5)		11 (40.7)	24 (70.6)	
Duration of ASM			0.923^a^			0.610^c^
< 1 year	15 (26.8)	16 (27.6)		2 (7.4)	3 (8.8)	
> 1 year	41 (73.2)	42 (72.4)		25 (92.6)	31 (91.2)	
Types of ASM			0.014^b^			0.472^b^
Traditional	17 (30.4)	28 (50.9)		11 (40.8)	14 (41.2)	
NewCombinations	12 (21.4) 27 (48.2)	14 (25.5) 13 (23.6)		8 (29.6) 8 (29.6)	14 (41.2) 6 (17.6)	
Number of ASM			0.001^a^			0.142^a^
Monotherapy	18 (32.1)	36 (62.1)		10 (27.1)	19 (55.9)	
Polytherapy	38 (67.9)	22 (37.9)		17 (62.9)	15 (44.1)	
Seizure Event			0.040^a^			0.061^a^
No	27 (48.2)	39 (67.2)		11 (40.7)	22 (64.7)	
Yes	29 (51.8)	19 (32.8)		16 (59.3)	12 (35.73)	

*ADR* Adverse drug reaction, *ASM* Anti-seizure medication

Chi-square test^a^

Likelihood ratio^b^

Fisher’s exact test^c^

Multivariate logistic regression analysis for the first period revealed that the incidence of ADRs was significantly associated with non-adherence (*P* = 0.007, OR: 5.216 [1.570–17.328]), and a higher number of ASMs was linked to non-adherence (*P* = 0.050, OR: 2.298 [1.001–5.277]) (Table 3).

**Table 3:** First period data: multivariate analysis of predictors associated with non-adherence

Step		Constant	*P*-value	OR	95% CI
Step 7b	ADRs (Yes)	1.652	0.007	5.216	1.570–17.328
	Number of ASM (Polytherapy)	0.832	0.050	2.298	1.001–5.277
	Constant	1.824	0.001	6.197	-

*ADR* Adverse drug reaction, *ASM* Anti-seizure medication, *OR* Odds ratio, *95% CI* (95% Confidence interval)

The mean knowledge score was 15.41 ± 3.827, while the median scores for attitude and behavior were 18 (10–27), and 10 (5–20), respectively (Table 4).

**Table 4:** KAB scores during second period data (*n* = 61)

Scores	Mean (SD) / Median (min-max)
Knowledge	15.41 (3.827)^a^
Attitude	18 (10–27)^b^
Behavior	10 (5–20)^b^

*SD* Standard deviation

Mean (SD)^a^

Median (min-max)^b^

**Fig. 3:**
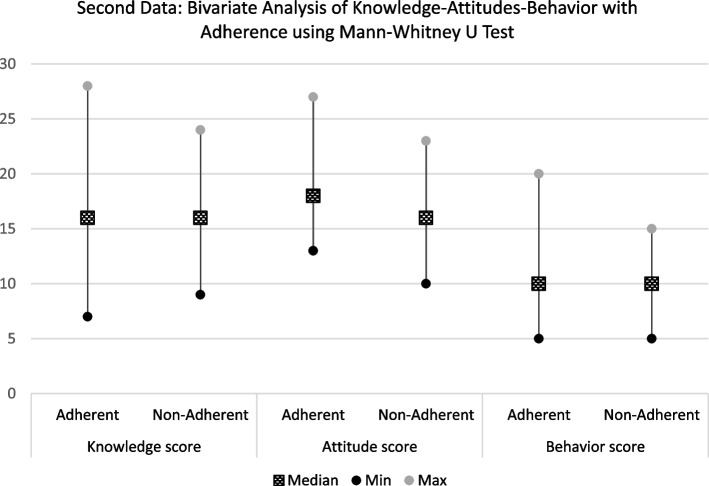
Second Data: Bivariate analysis of KAB with adherence (Knowledge-Adherence *P* = 0.426; Attitudes-Adherence *P* = 0.061; Behavior-Adherence *P* = 0.130)

Although knowledge and behavior did not differ significantly between adherent and non-adherent groups, the minimum knowledge score in the adherent group was lower, indicating better overall knowledge level. Interestingly, the attitude scores of adherent subjects were poorer, with a higher median value compared to non-adherent subjects (Fig. 3, Table 5).

**Table 5:** Second period data: bivariate analysis of KAB with adherence

	Adherence		*P*-value
	Adherent (*n* = 34)	Non-adherent (*n* = 27)	
Knowledge score (median [min–max])	16 (7–28)	16 (9–24)	0.426^a^
Attitude score (median [min–max])	18 (13–27)	16 (10–23)	0.061^a^
Behavior score (median [min–max])	10 (5–20)	10 (5–15)	0.130^a^

*SD* Standard deviation

Mann–Whitney U test^a^

Only gender (*P* = 0.037) was found to have a significant association with adherence in the second period (Table 2). The multivariate analysis included variables with a *P*-value < 0.1, such as knowledge, gender, ADRs, attitude, and behavior. Logistic regression showed a significant association between gender and adherence, with female subjects being 5.404 times more likely to be adherent (*P* = 0.013, OR: 0.185 [0.049–0.704]). Additionally, poorer knowledge (higher scores) was associated with higher odds of non-adherence (*P* = 0.018, OR: 1.271 [1.043–1.550]) (Table 6).

**Table 6:** Second period data: multivariate analysis of ADRs incidence and KAB to non-adherence level of epileptic patients

Step		Constant	*P*-value	OR	95% CI
Step 1a	Knowledge	0.240	0.018	1.271	1.043–1.550
	Attitude	−0.191	0.082	0.826	0.666–1.025
	Behavior	−0.141	0.276	0.869	0.675–1.119
	Gender (Female)	−1.687	0.013	0.185	0.049–0.704
	ADRs (Yes)	1.640	0.109	5.155	0.696–38.198
	Constant	−0.243	0.907	0.784	-

*ADR* Adverse drug reaction, *KAB* Knowledge, attitude, behavior, *OR* Odds ratio, *95% CI* 95% Confidence interval

**Fig. 4:**
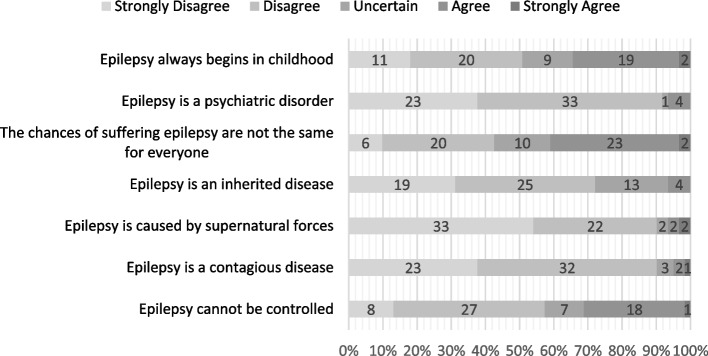
Second Data: Distribution of answers for knowledge statements (All Subjects, *n* = 61)

**Fig. 5:**
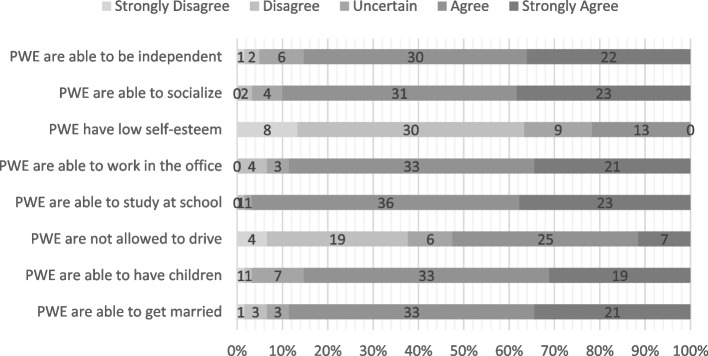
Second Data: Distribution of answers for attitude statements (All Subjects, *n* = 61)

**Fig. 6:**
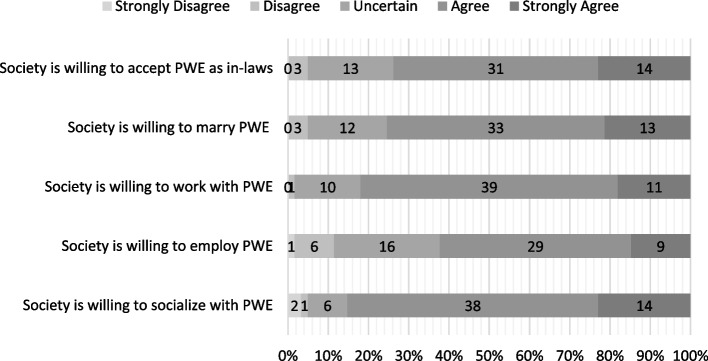
Second Data: Distribution of answers for behavior statements (All Subjects, *n* = 61)

The responses for items of the knowledge-attitudes-behavior questionnaire are displayed in Figs. 4, 5 and 6, respectively. A sub-analysis of items in each group (KAB) was conducted by merging “strongly disagree” and “disagree” responses into one “disagree” response, with the same pattern of merging being done for the “agree” responses, thereby giving way to only three categorical responses: disagree, uncertain, agree. In the knowledge section, there might be a correlation between the item regarding the likelihood of suffering from epilepsy and adherence (*P* = 0.042), with a higher percentage of subjects in the non-adherent group answering this item correctly (53.8% vs 34.3%). Moreover, subjects from the non-adherent group had better attitudes on driving prohibitions, and had better perceptions regarding society’s behavior in accepting them. However, they reported lower self-esteem (Table 7).

**Table 7:** Second period data: breakdown of KAB items and association towards adherence

Items	Adherent (*n*%)	Non-Adherent (*n*%)	*P*-value
**Knowledge**			
Epilepsy always begins in childhood			0.667^a^
· Disagree	19 (54.3)	12 (46.2)	
· Uncertain	4 (11.4)	5 (19.2)	
· Agree	12 (34.3)	9 (34.6)	
Epilepsy is a psychiatric disorder			0.397^a^
· Disagree	33 (94.3)	23 (88.5)	
· Uncertain	0 (0)	1 (3.8)	
· Agree	2 (5.7)	2 (7.7)	
The chances of suffering epilepsy are not the same for everyone			**0.042**^a^
· Disagree	12 (34.3)	14 (53.8)	
· Uncertain	4 (11.4)	6 (23.1)	
· Agree	19 (54.3)	6 (23.1)	
Epilepsy is an inherited disease			0.392^a^
· Disagree	26 (74.3)	18 (69.2)	
· Uncertain	8 (22.9)	5 (19.2)	
· Agree	1 (2.9)	3 (11.5)	
Epilepsy is caused by supernatural forces			0.138^a^
· Disagree	32 (91.4)	23 (88.5)	
· Uncertain	0 (0)	2 (7.7)	
· Agree	3 (8.6)	1 (3.8)	
Epilepsy is a contagious disease			0.887^a^
· Disagree	31 (88.6)	24 (92.3)	
· Uncertain	2 (5.7)	1 (3.8)	
· Agree	2 (5.7)	1 (3.8)	
Epilepsy cannot be controlled			0.482^a^
· Disagree	21 (60)	14 (53.8)	
· Uncertain	5 (14.3)	2 (7.7)	
· Agree	9 (25.7)	10 (38.5)	
**Attitude**			
PWE are able to be independent			0.329^a^
· Disagree	2 (5.7)	1 (3.8)	
· Uncertain	5 (14.3)	1 (3.8)	
· Agree	28 (80)	24 (92.4)	
PWE are able to socialize with other people			0.738^a^
· Disagree	1 (2.9)	1 (3.8)	
· Uncertain	3 (8.6)	1 (3.8)	
· Agree	31 (88.6)	24 (92.3)	
PWE have low self-esteem			0.621^a^
· Disagree	24 (68.6)	15 (57.7)	
· Uncertain	5 (14.3)	4 (15.4)	
· Agree	6 (17.1)	7 (26.9)	
PWE are able to work in the office			0.697^a^
· Disagree	3 (8.6)	1 (3.8)	
· Uncertain	2 (5.7)	1 (3.8)	
· Agree	30 (85.7)	24 (92.3)	
PWE are able to study at school			0.244^a^
· Disagree	1 (2.9)	0 (0)	
· Uncertain	0 (0)	1 (3.8)	
· Agree	34 (97.1)	25 (96.2)	
PWE are not allowed to drive			0.753^a^
· Disagree	14 (40)	9 (34.6)	
· Uncertain	4 (11.4)	2 (7.7)	
· Agree	17 (48.6)	15 (57.7)	
PWE are able to have children			0.244^a^
· Disagree	2 (5.7)	0 (0)	
· Uncertain	3 (8.6)	4 (15.4)	
· Agree	30 (85.7)	22 (84.6)	
PWE are able to get married			0.697^a^
· Disagree	3 (8.6)	1 (3.8)	
· Uncertain	2 (5.7)	1 (3.8)	
· Agree	30 (85.7)	24 (92.3)	
**Behavior**			
Society is willing to accept PWE as in-laws			0.222^a^
· Disagree	2 (5.7)	1 (3.8)	
· Uncertain	10 (28.6)	3 (11.5)	
· Agree	23 (65.7)	22 (84.6)	
Society is willing to marry PWE			0.089^a^
· Disagree	2 (5.7)	1 (3.8)	
· Uncertain	10 (28.6)	2 (7.7)	
· Agree	23 (65.7)	23 (88.5)	
Society is willing to work with PWE			0.329^a^
· Disagree	1 (2.9)	0 (0)	
· Uncertain	7 (20)	3 (11.5)	
· Agree	27 (77.1)	23 (88.5)	
Society is willing to employ PWE			0.162^a^
· Disagree	6 (17.1)	1 (3.8)	
· Uncertain	10 (28.6)	6 (23.1)	
· Agree	19 (54.3)	19 (73.1)	
Society is willing to socialize with PWE			0.289^a^
· Disagree	1 (2.9)	2 (7.7)	
· Uncertain	2 (5.7)	4 (15.4)	
· Agree	32 (91.4)	20 (76.9)	

## Discussion

Our findings suggested that ASM adherence was significantly associated with the incidence of ADRs in the first period, and with knowledge scores in the second period.

In the first period of our data, women exhibited higher adherence rates, but not to a significant extent. However, in the second period, this difference became significant. This discrepancy may be partly due to the larger number of variables collected and the more detailed statistical adjustments made during the second period. These changes likely helped to identify associations that were not apparent in the first period. Notably, previous studies have similarly reported that women are more likely to adhere to treatment than men [18–21]. This phenomenon may be due to several factors, including the more proactive health-seeking behaviors and frequent medical consultations observed among women [22]. Additionally, women often benefit from stronger social support networks, which play a critical role in encouraging medication adherence [23].

Furthermore, higher adherence was observed among patients who did not report seizure events. This association was apparent in bivariate analysis, but was not significant in multivariate analysis. Other reports have equally found that poor seizure control or higher seizure frequency was significantly linked to non-adherence [18, 24, 25].

### Medication adherence in PWE

The MAQ is a practical tool to assess medication adherence, consisting of only four straightforward questions, making it convenient for patients [11, 12]. At the beginning of this study, we observed that most of the subjects were non-adherent. Between the first and second periods of data collection, several interventions were introduced to enhance adherence, including health promotion through social events and social media, as well as increased patient education in routine clinical practice. In the second period, conducted approximately three years later, we observed a higher adherence rate. While this improvement may reflect the potential benefits of these initiatives, it is important to acknowledge that the time gap coincided with major external disruptions, including the COVID-19 pandemic, which we did not account for. During this time, changes in the daily routines of PWE may have influenced adherence. For instance, spending more time at home could have provided them with greater opportunities to learn about epilepsy through online resources such as webinars. However, we did not assess or measure these behavioral and educational changes, as well as other factors like shifts in the healthcare system, limiting our ability to directly attribute the observed improvement to specific interventions.

### Anti-seizure medication ADR in PWE and its association with medication adherence

We found that 78.1% of PWE in the first period of our study experienced ADRs, while 82% reported ADRs in the second period. A previous study in 2018 by Budikayanti et al. reported an even higher ADR incidence of 91% among PWE at CMH [14]. Similarly, studies by Carreno et al. and Canevini et al. found high ADR incidences of 65.2% and 96%, respectively. The method used to detect ADRs plays a critical role in these findings. Questionnaires tend to identify more ADRs compared to spontaneous patient reporting, likely because patients may be unaware that their symptoms are related to ASMs or may feel hesitant to report them. Additionally, time constraints during consultations may prevent clinicians from inquiring about all possible ADR symptoms, leading to underreporting [26, 27].

The most commonly reported ADRs in both periods of our study were sleepiness, memory problems, headaches, and irritability. In the first period, difficulty in concentration and unsteadiness were frequently observed, while in the second period, tiredness and nervousness were more common. Similar findings were reported in studies by Budikayanti et al., Baker et al., Chen et al., and Martins et al. [13, 14, 28, 29]. The primary mechanism of action of ASMs is to reduce neuronal hyperexcitability, which underlies seizures. As a result, the most frequently reported ADRs are typically related to the central nervous system, reflecting the impact of ASMs on brain function [29].

Most studies on ASM-related ADRs and medication adherence have been conducted on general populations of PWE, without focusing on specific types of epilepsy. Consistent with these findings, our study also identified a significant association between ADRs and ASM adherence during the first period; patients who reported ADRs had 5.2 times the odds of being non-adherent. This aligns with prior research, such as Tan et al.'s study in Malaysia, which found that 32.3% of PWE with ADRs had low adherence [5]. Getnet et al. reported a higher incidence of non-adherence (50%) and noted that the majority of complaints from PWE were related to drug side effects, which were often cited as the primary reason for discontinuing ASM without consulting a healthcare provider [6]. However, the larger sample size in the first period may have increased statistical power, potentially influencing the detection of significant associations. Therefore, these findings should be interpreted with caution, as the observed significance might reflect sample size effects rather than true temporal changes.

In the bivariate analysis of our first dataset, both the type and number of ASMs prescribed, along with the presence of ADRs, were significantly associated with medication adherence in PWE (*P* < 0.05). However, multivariate analysis revealed that only the number of ASMs remained significantly associated with adherence (*P* = 0.05). Our study found that patients on monotherapy were more likely to be adherent compared to those on polytherapy. Additionally, an association was observed between polytherapy and a higher frequency of reported ADRs. This observation aligns with findings from studies in other countries [28–31]. A study by Joshi et al. in India noted no direct association between ASM types and ADRs; however, patients on combinations of traditional and new ASMs had higher LAEP scores. Several combinations were associated with high LAEP scores above 30, including carbamazepine with lamotrigine; carbamazepine, lamotrigine, and topiramate; carbamazepine, valproate, and clobazam; carbamazepine, clobazam, and topiramate; as well as phenytoin with valproate and lamotrigine [32].

Drug interaction was one of the underlying possible explanations on the occurrence of ADR in polytherapy. Pharmacokinetic interactions that occur due to the effect of one drug on another would cause changes in the absorption, metabolism, protein binding, and excretion, thus affecting one’s drug efficacy as well as increasing ADR risk [33]. The use of combination of traditional and new ASMs were intended to avoid drug interactions [34]. However, in this study, the most commonly used new generation drugs were metabolized in the liver, which were acting as a substrate (lamotrigine) or an inducer/inhibitor (topiramate). They interacted with traditional ASMs which are extensively metabolized in the liver as enzyme inducers or inhibitors. The combination of carbamazepine and lamotrigine interacts majorly both pharmacodynamically and pharmacokinetically. Carbamazepine induces lamotrigine metabolism, thus reduced its levels (although in some studies lamotrigine levels in blood did not change significantly). Lamotrigine can potentially increase blood levels of the epoxide carbamazepine metabolite, which can lead to neurotoxicity. Pharmacodynamically, these two types of ASMs have the same site of action, namely the sodium channel. The combination of ASMs with the same mechanism of action can increase the risk of decreased patient tolerability and a higher risk of neurotoxicity [35].

The combination of valproate and lamotrigine pharmacokinetically interacts in a significant way [35]. Valproate, a glucuronidation inhibitor, can increase lamotrigine levels in plasma and increase the risk of lamotrigine-related ADRs. In this study, valproate and lamotrigine that were used in combination with phenytoin had relatively high LAEP scores of 31. Drug interaction between phenytoin and valproic acid leads to the displacement of phenytoin from plasma proteins. It inhibits phenytoin metabolism, thereby increasing serum concentrations of free drug (phenytoin) and elevating the risk of developing phenytoin toxicity. Another combination of carbamazepine, clobazam, and topiramate, also showed high LAEP scores ranged from 26 to 33. At the time of the interview, the patient complained about restlessness and sleepiness with frequent intensity, resulting in impairment of daily activities and productivity. Pharmacokinetic interactions are also observed between clobazam and topiramate; hence when these two drugs are combined, the risk of sedation would be increased (additive CNS depression) [35].

The adherence rate was lowest (23.6%) among subjects taking a combination of both traditional and new ASMs, compared to those prescribed only traditional or new ASMs alone (Table 2). This indicates an association between combination ASM therapy and lower adherence. Our findings align with Das et al., who observed that adherence decreases as the number of medications increases. This is most likely due to the complexity of managing multiple drugs, including increased side effects and potential drug interactions [36].

Poorly managed ADRs not only increase the risk of seizure recurrence but also contribute to reduced quality of life and heightened anxiety regarding ASM use [5]. Treating physicians should adopt structured counseling strategies that focus on recognizing, managing, and minimizing ADRs while reinforcing the importance of adherence. Patient education should include discussions on common ADRs, their expected duration, and when to seek medical advice. Shared decision-making, where treatment adjustments are discussed collaboratively, can also improve patient engagement and adherence. Meanwhile, routine follow-ups can assess patients’ experiences with ASMs and allow for dosages adjustments or medication switches if side effects become intolerable. The implementation of a “side-effect diary” in relation to certain ASMs could further enhance ADR documentation and adherence monitoring [37].

### KAB associated with medication adherence in PWE

A previous study found that KAB of PWE at CMH were generally positive, leading us to anticipate higher adherence rates [15]. However, adherence remained lower than expected, particularly when compared to other similar studies [6, 8, 9]. This suggests that additional factors may influence adherence beyond just positive KAB. Common factors such as age, gender, treatment costs, regimen complexity and ADR are often thought to impact adherence, but our multivariate analysis revealed that knowledge and gender were independently associated with adherence. Specifically, women were more likely to adhere to their medication regimen as mentioned before.

Knowledge in this study was assessed with 7 questions about general knowledge concerning epilepsy, including false myths, the chances of suffering from epilepsy, pathogenesis, and prognosis. Interestingly, while non-adherent subjects debunked common myths and had better knowledge about the likelihood of developing epilepsy, nearly half of both adherent and non-adherent participants believed seizure freedom was unachievable, particularly within the non-adherent group. This disbelief in achieving seizure control may explain their non-adherence, a finding echoed by Nakhutina et al., who reported that PWE were more likely to have low adherence when they doubted the effectiveness of their medication in achieving seizure freedom [38]. A study in India demonstrated a strong link between knowledge of epilepsy and treatment adherence, finding that non-adherent PWE had minimal understanding of the disease and its therapy [39]. Patients’ awareness and perceptions regarding the importance of epilepsy treatment significantly impacted adherence, with those displaying low adherence also perceiving a reduced need for medication [40].

Most subjects displayed positive attitudes, with those in the non-adherent group showing greater self-awareness regarding their ability to drive. While countries like the United States have established clear guidelines, such as those from the American Academy of Neurology and the American Epilepsy Society, Indonesia has yet to implement specific driving regulations for PWE. A study in Australia found that 52% of crashes involving drivers with epilepsy were seizure-related, often involving a single vehicle colliding with a fixed object, and that PWE were 1.13 to 2.16 times more likely to experience serious accidents compared to non-epileptic drivers [41, 42]. Although the non-adherent group displayed relatively better attitudes about driving, they also reported lower self-esteem, a common issue among PWE. This suggests that, besides attitude, psychological aspects could also play a role in shaping adherence. A study in Iran confirmed that low self-esteem is directly associated with reduced adherence to medication. Higher self-esteem enhances medication adherence because individuals with greater self-worth are more likely to believe in their ability to manage their health, adopt positive behaviors, and maintain consistency in following treatment plans [43].

Most subjects also exhibited positive behaviors. Interestingly, nearly all individuals in the non-adherent group believed that society would accept them as in-laws, potential partners, colleagues, or employees. Recent studies from Saudi Arabia, China, and Indonesia reported marriage rates among PWE at 47%, 62%, and 40.8%, respectively [15, 44, 45]. Similarly, a systematic review showed a global mean employment rate of 58% for PWE, while Indonesia’s employment rate for this group was 37.4% [15, 46]. Despite this perceived acceptance, many PWE were not confident in their place within society. Previous research indicated that while Indonesian society is generally reluctant to form deeper bonds with PWE, it still demonstrates favorable behavior toward them [15]. A study exploring the relationship between planned behavior and medication adherence in epilepsy found that while attitude and behavioral intention influenced adherence, the subjective norm—defined as societal perception—did not. This could indicate that PWE’s adherence behaviors are shaped more by personal factors than by how they believe society views them [47].

In our study, neither attitude nor behavior were significantly associated with medication adherence. In fact, the non-adherent group even scored slightly higher than the adherent group in certain KAB domains. However, they scored significantly lower on the item assessing their perceived likelihood of developing epilepsy. This lower perception of risk may reduce their belief in the importance of medication. In Indonesia, many individuals attribute epilepsy to fate, which can lead to the perception that treatment has little influence on disease outcomes. By addressing this misconception—emphasizing that epilepsy can affect anyone, and that it can be effectively managed through ASMs—we predict that adherence rates can be improved.

Another possible reason why neither attitude nor behavior showed a significant association with medication adherence could be the relatively small sample size. Additionally, the questionnaire items may have been more reflective of quality of life (QoL) rather than directly measuring factors influencing medication adherence. This could have limited the ability to detect a clear relationship between attitude, behavior, and adherence. Studies that have found correlations between attitude, behavior, and adherence often use tools like the Beliefs about Medicine Questionnaire (BMQ) or other instruments designed to assess beliefs specifically related to medication [48, 49]. In particular, Dayapoglu et al., found that negative beliefs among PWE, such as concerns about medication side effects and doubts about the purpose of the medication, were linked to lower adherence rates [50]. A similar finding was replicated in Ethiopia where low medication necessity belief, high medication concern belief, and overall negative medication belief were correlated to an increase in non-adherence rates [10].

On the other hand, another study in Ethiopia indicated that knowledge about epilepsy and its treatment had a stronger influence on adherence than attitude. Comparable to our study, the authors also devised their own questionnaire regarding general knowledge and attitude on epilepsy based on previous studies. This suggests that even if individuals display positive attitudes, they may not necessarily adhere to ASMs. Patients with greater knowledge of epilepsy and its treatment are more likely to adhere, as they better understand the risks of non-adherence and the importance of consistent medication use [51].

Other factors such as adverse drug reactions, forgetfulness, or complex medication schedule, namely subjects on polypharmacy, can also prevent adherence [52]. This was evident in our study as our results yielded many subjects who were on polytherapy, or experienced adverse drug reactions, as being less adherent. Some patients may have positive attitudes but believe they can skip doses without serious consequences, leading to non-adherence despite their behavior and attitudes suggesting otherwise​. Additionally, patients might exhibit positive attitudes and behaviors during clinical assessments, but cognitive issues like memory lapses or emotional factors like depression can still result in poor adherence​ [53, 54]. Unfortunately, we did not assess the mental state, or account for the screening of depression in this study.

### Limitations and future implications

One strength of this study was the use of the self-reported LAEP questionnaire, which allowed for the capture of patients’ specific complaints, providing valuable insights into their personal experiences with ASMs. Additionally, the study was conducted at a national referral hospital, offering a diverse representation of ASM patient characteristics and treatments, making the findings more reflective of the broader epilepsy population in Indonesia. However, there were some limitations. The reliance on self-reported medication adherence without objective measures such as therapeutic drug monitoring may have introduced recall or reporting bias. Furthermore, the relatively small sample size may have affected the statistical power of our results, limiting the generalizability of the findings. Although we controlled for certain confounding factors, such as age, gender, duration of ASM use, type and number of ASMs, and seizure events, other potential confounders, particularly time-related confounders, were not documented. Notably, all participants were treated under the national health insurance system, minimizing variations in healthcare access, but we did not collect data on socioeconomic status, cultural background or religiosity, which could influence adherence behaviors. This was also the reason why we could not perform ideal stratification analysis of the two periods for comparison. Another limitation of our study is that it combined data from two independent cross-sectional studies conducted three years apart. Although the research questions and methods were aligned from the outset, the temporal gap introduces the possibility of unmeasured changes over time, including differences in healthcare system, and patient demographics—particularly given the intervening COVID-19 pandemic. Additionally, due to Indonesia’s national referral policy, many patients from the first period who had achieved seizure control were referred back to their primary care centers. As a result, the two study periods involved different patient cohorts, limiting our ability for direct comparison. Although the study was hypothesis-driven, the cross-sectional design of the study limited our ability to determine causality.

Future research using tools specifically designed to assess KAB toward medication adherence, incorporating objective adherence measures such as drug level monitoring, and including socioeconomic background as confounding factors would help strengthen and expand upon these findings.

## Conclusions

While our study provides valuable insights into factors influencing ASM adherence among PWE, its limitations should be considered when interpreting the findings. Certain confounding factors such as socioeconomic status, spirituality, cultural background and other time-related confounders were not accounted for. Moreover, the absence of therapeutic drug monitoring, and relatively small sample size may have introduced biases, potentially affecting the accuracy of adherence estimates and limiting generalizability.

Even so, we revealed a significant association between the incidence of ADRs and medication adherence, with PWE who reported ADRs being more likely to be non-adherent. Other factors significantly associated with adherence included the number of ASMs, gender, and knowledge. Notably, patients on monotherapy and females were observed to be more adherent. Higher knowledge scores were related to better adherence, and patients with greater knowledge were also more likely to report ADRs. These findings suggest that knowledge may influence both adherence and symptom awareness, though causality cannot be inferred due to the cross-sectional design. Meanwhile, non-adherent subjects demonstrated greater awareness regarding driving and more positive perceptions of societal acceptance but exhibited lower knowledge regarding the chances of suffering from epilepsy.

To improve adherence, patients experiencing ADRs should be given individualized medication counselling and symptom management strategies to mitigate side effects without discontinuing treatment. For those on polytherapy, careful medication reviews and dose adjustments should be conducted to minimize drug interactions and adverse effects. Healthcare providers are recommended to provide structured educational programs to increase epilepsy-related knowledge; focusing on the importance of ASM adherence, and potential ADRs, while also considering psychological aspects of the patient, and their respective knowledge, attitudes, and behaviors. Future research should explore the relationship between drug availability, KAB, and ASM adherence in a larger population to provide deeper insights into adherence behaviors and develop more effective interventions.

## Data Availability

Due to the sensitive nature of the disease, data regarding subjects are strictly confidential, and will not be shared. More information concerning research data is available on request to the corresponding author.

## Abbreviations

ADRs: Adverse drug reactions
AI: Artificial intelligence
ASM: Anti-seizure medications
BMQ: Beliefs about Medicine Questionnaire
CMH: Cipto Mangunkusumo National Referral Hospital
DRE: Drug-resistant epilepsy
MAQ: Morisky Adherence Questionnaire
KAB: Knowledge-attitude-behavior
PWE: Patients with epilepsy
QoL: Quality of life
